# COVID-19 and mental health of older adults in the Philippines: a perspective from a developing country

**DOI:** 10.1017/S1041610220000757

**Published:** 2020-04-30

**Authors:** Robert D. Buenaventura, Jacqueline B. Ho, Maria I. Lapid

**Affiliations:** 1 Department of Psychiatry, Manila Theological College – College of Medicine, Manila, Philippines; 2 University of California Berkeley, College of Letters & Science, Berkeley, CA, USA; 3 Mayo Clinic, Department of Psychiatry and Psychology, Rochester, MN, USA

## Introduction

As the world grapples with the public health emergency and economic crisis from the coronavirus disease 2019 (COVID-19) pandemic, developing countries become the most vulnerable to its profound negative impact. The current struggles of developed countries in responding to the pandemic despite their wealth of resources, solid economies, and established healthcare infrastructures are magnified and intensified for developing countries. Challenges of meeting even the most basic health needs in developing countries become much more acute in the setting of a pandemic, and physical health takes precedence over mental health needs. In this commentary, we discuss the impact of COVID-19 on the mental health of older Filipinos, who are more vulnerable to the effects of COVID-19, and describe ways that healthcare workers can help alleviate the negative impact on their mental health to the extent possible within the limited resources available in the Philippines.

## Country-specific factors that increase vulnerability to COVID- 19

Developing countries are stratified according to income levels, which likely predict a country’s ability to mount a response to a pandemic. Even among developing countries, there are certain challenges that are unique to each country in managing the COVID-19 pandemic. We explore some of the factors characteristic to the Philippines that increase the country’s vulnerability to the negative effects of COVID-19.

### High population density

The Philippines is an archipelago in Southeast Asia with a population of over 100 million, of which 60% is concentrated in the main island of Luzon, and the rest make up the other two main islands Visayas and Mindanao which are mostly rural areas. The Philippine population density is 337 persons per square kilometer, although in the National Capital Region (NCR) alone in Luzon, the population density is a staggering 20,785 persons per square kilometer (Philippine Statistics Authority, 2016b). To put this in a perspective, the United States in comparison has a population density of about 90 people per square mile, and although density values vary from one city to another, most people live in cities with about 1600 people per square mile (Darryl, 2015). Since the spread of COVID-19 is related to population density (Rocklov and Sjodin, 2020), the high densities in the Philippines where people are in relatively close contact with each other in both personal and public spaces make social distancing difficult, facilitate virus spread more easily, and lead to higher rates of infection and death. In fact, within Southeast Asia, the Philippines has the highest number of coronavirus infection, with a mortality of 40% (Lema and Morales, 2020).

### Demographics and social welfare

Adults 65 years and older and those with underlying medical conditions such as cardiovascular disease, hypertension, and diabetes are at higher risk for developing more serious complications from COVID-19 (Applegate and Ouslander, 2020; Rocklov and Sjodin, 2020). In the Philippines, older individuals comprise 7.5% of the total population equivalent to 7.5 million individuals (Philippine Statistics Authority, 2016a). Older Filipinos have a life expectancy of 71 years, which is slightly below the world average of 72 years (Philippine Statistics Authority, 2016a). Of the 7.5 million senior citizens in the country, only 30% receive social pension of P500 monthly (USD$10) and 40% receive no pension at all (Senate of the Philippines, 2019). In the context of a pandemic, the economic insecurity of older Filipinos heightens their vulnerabilities in terms of inability to access health care and pay for healthcare bills (Badana and Andel, 2018). Older Filipinos who live in rural areas have more difficulties with access to healthcare services than those in urban settings. The high mortality rate of 40% from COVID-19 disproportionately affects the older Filipinos. As of April 15, 2020, there are 5453 confirmed COVID-19 cases and 349 deaths, of which 50.7% are aged 65 years and older (Department of Health, 2020). Overall, it is not just advanced age and medical comorbidities that make older Filipinos vulnerable to COVID-19, but poverty also plays a significant role in their high death rates.

### Cultural habits

The Philippines ranks as the third highest Catholic population in the world after Brazil and Mexico (World Population Review, 2020). Majority of older Filipinos are deeply religious and have strong views of God’s role in health and well-being. The strong faith and spirituality of older Filipinos serve as a strength and protective buffer to stress and suffering (Esteban, 2015). Going to church regularly and attending church activities and gatherings are part of everyday life for older Filipinos. Churches in the Philippines are usually packed and overcrowded, with people sitting very close to each other in an enclosed space with decreased ventilation and poor air circulation. Mass in church involves singing and praying together, shaking hands when greeting each other with peace, receiving communion served by the priest or Eucharistic ministers, and drinking wine from a communal cup. All of these factors facilitate viral spread and transmission so being in a church and attending mass and other gatherings can be deadly for older Filipinos who are especially vulnerable to the disease.

Another unique tradition in the Philippine culture is the concept of multigenerational households, where multiple generations of families including grandparents, parents, children, and grandchildren live under one roof. It is difficult to practice social distancing within the home, or separate children from grandparents. There is a potential for family members to be carriers and expose older family members or cause them to get infected. In poor households, it is impossible to implement social distancing.

### Health systems capacity

Health systems need to be mobilized in unprecedented ways to mitigate the impact of the COVID-19 outbreak, and it is unclear whether health systems in developing countries will be able to cope. Health services in the Philippines are delivered through public (Department of Health) and private sectors (for-profit and nonprofit healthcare providers) (Dayrit *et al.*, 2018), although funding and insurance are limited, and majority of Filipinos pay out of pocket for healthcare expenses. Becoming ill and needing treatment or hospitalization are extremely expensive, and medical costs are prohibitive for an average Filipino. While advances have been made to improve the fragmented healthcare system in the Philippines, continued inequities in health care significantly affect the poor. Currently, the hospitals in the country are overwhelmed and reaching maximum capacity, with severe shortage of beds, lack of ability to conduct COVID-19 testing, and inadequate supply of personal protective equipment. The poor healthcare infrastructure in the country makes it ill prepared to respond to the COVID-19 pandemic.

## The impact of COVID-19 outbreak on the mental health of older Filipinos

The first COVID-19 case in the Philippines was reported on January 30, 2020, although it was not until over a month later that the country was placed under a state of public health emergency. Subsequently, the country was placed on enhanced community quarantine (ECQ) which is effectively a total lockdown for two months to limit movement of people. All local government units are allowed to impose their own version of ECQ which involves travel restrictions (air, sea, and land), suspension of nonessential work, prohibition of mass gatherings, and closure of establishments except those providing or manufacturing basic necessities. Some local governments have gone to the extent of imposing curfews and arrests for those who violate curfews and lockdown.

Across the world countries are currently in some degree of “lockdown” that requires people to stay at home and maintain social distance in order to avoid catching or spreading COVID-19 and break the chain of transmission. It is unclear yet whether the Philippine lockdown is working as the health system in the country is further strained with continued growing crisis. While the lockdown and other public health measures are designed to keep individuals from getting infected with COVID-19, there are unintended consequences on the mental health of older individuals who are at most risk and in whom the strictest quarantine measures apply.

### Depression and anxiety

The onset of a new pandemic for which people had no prior experience in dealing with has wrought fear on the population, particularly for older people because they are considered at higher risk for the disease. This has led them to be housebound, venturing out only when extremely necessary. It is not easy to cope with prolonged confinement, and amidst mandated isolation, seniors may experience depressive symptoms, loneliness, pessimism, deteriorations in cognition, and disruption in sleeping patterns (Avasthi and Grover, 2018). These are consistent with the known psychological reactions of stress, anxiety, loneliness, and agitation in a pandemic (Meng *et al.*, 2020).

Social connectedness and engagement with other people are important to promote successful ageing; however, this being directly challenged by physical distancing policies (Brauser, 2020; Williams, 2020). A survey showed that 37.1% of elderly amidst the COVID-19 pandemic in China showed significant depression and anxiety (Qiu *et al.*, 2020). The disruption of important day-to-day activities for older individuals can pose negative impact on elderly’s cognitive impairment, leading to poorer mental health, low quality of life (Santini *et al.*, 2020), and anxiety (World Health Organization, 2020). During quarantine, persistent symptoms include regular and debilitating worry about routine activities (Subramanyam *et al.*, 2018), and lack of social events and support also heighten stress and decrease coping skills (Avasthi and Grover, 2018); therefore, older individuals tend to fixate on the uncertainty of this pandemic and feel agitated (Huang and Zhao, 2020). Overall, community quarantine poses symptoms associated with late-life anxiety and depression.

### Unmet spiritual needs

The quarantine period overlapped with the Roman Catholic Lenten Season, an extremely important holiday for Filipinos. Older Filipinos were unable to attend church services and observe traditional Holy Week religious practices thereby increasing their sense of isolation. For many older Filipinos going to the church every Sunday followed by family gatherings is a tradition that they look forward to but is now not possible, and this has added to a sense of sadness and yearning. Older Filipinos have also lost connection with their spiritual leaders, church peers, and volunteer work which may exacerbate feelings of uncertainty.

### Poor social well-being

Older Filipinos who at baseline already experience loneliness and have no moral support at home face more challenges. Even though physical distancing is critical to reduce the spread of the virus, the result is that many older individuals are now unable to see friends and family and experience further isolation. Those who do not have access to technology and have very limited resources are often much more isolated and unable to connect with others outside of the home. This lack of social interaction can lead to long-term distress and a decline in emotional well-being; this effect is more prominent especially among geriatrics who have limited fluency with digital platforms (Newman and Zainal, 2020). The regular social support older adults relied on may be diminished. The restrictions from the community quarantine have also affected their mobility, including those who are self-sufficient, as they are prohibited from going out and barred from public places such as groceries and supermarkets. There are reports of older Filipinos breaking quarantine to make a living to support themselves because they have no one else to depend on or they did not want to be a burden to younger family members.

### Decline in physical well-being

A benefit of quarantine is keeping people safe at home; however, a health risk with being at home is a decline in physical activity which has a negative impact on older individuals. Staying at home leads to a more sedentary lifestyle such as sitting a lot or spending more time watching TV and engaging in unhealthy behaviors such as overeating, staying up late and not getting adequate sleep, or even increasing tobacco and alcohol use. Reduced physical activity and unhealthy behaviors are linked to increased morbidity and mortality in older individuals (Schrempft *et al.*, 2019). A significant challenge during prolonged lockdowns is to minimize the adverse effects of physical inactivity on older individuals. In addition, the lockdown has resulted in older Filipinos having very limited or no access to healthcare services since almost all outpatient clinics are closed because healthcare resources are mostly diverted to emergent care settings. Older Filipinos who frequently have chronic medical conditions find themselves worrying about their futures and praying that nothing unexpected happens during the lockdown.

### Dying alone

Patients with COVID-19 who are hospitalized are completely isolated in the ward and separated from their loved ones as no visitors are allowed in the hospital. Older Filipinos in the hospital with little capability of using digital technology have a difficult time adjusting to the isolation and minimal contact with their families. When they clinically deteriorate, it becomes extremely difficult to be alone, and particularly in the worst outcomes when a patient dies alone in the hospital. This is extremely difficult to cope with, as nobody should die alone, and families who are unable to see their loved ones before they die are at risk of complicated bereavement. Another aggravating factor for families left behind is the restriction on wakes and burials, which are traditionally week-long events filled with religious and ceremonial activities in the Philippines, often leading to lack of closure over the death of their loved ones.

## Management strategies to help maintain well-being of older Filipinos

Approaches to helping mitigate the negative impact of the COVID-19 pandemic on older Filipinos involve interventions with emotional, spiritual, social, and physical components to meet their mental and other health needs.

For anyone who is experiencing emotional distress, the National Center for Mental Health provides a crisis hotline that is free to the public. Providers in the private sector and nongovernment organizations pitched in as well, offering free telephone and online consultation and counseling services. Through telehealth measures, older individuals with anxiety and depression can benefit from psychotherapeutic treatments such as cognitive behavioral therapy (CBT) (Subramanyam *et al.*, 2018) to correct maladaptive behavior and negative thoughts of distress and hopelessness (Diefenbach and Goethe, 2006). For physical and medical concerns, the public have access to the Department of Health hotline for free telemedicine consultation services for any medical conditions. For the older Filipinos who have anxieties about their physical health, local government units (LGUs) created a Barangay Health Emergency Response Team (BHERT) that serves as a triage unit prior to referral to a hospital or a designated COVID-19 medical center. These triage units are more convenient for older individuals to access given the locations are local within their area of residence. The BHERT can also dispatch a service vehicle to assist elderly patients in need.

Cognizant of the challenges that older Filipinos face, LGUs have been tasked to assist them whenever possible. Communities have stepped up to help out as well, including buying basic commodities for the older neighbors. Social groups likewise volunteered to look in on older Filipinos who live alone to assess their needs and to ensure they are managing on their own. The national government also considers older Filipinos as a top priority in its social amelioration program of financial assistance during the pandemic together with programs initiated by other government officials and nongovernment organizations. Family caregivers have been helping to maintain social connections and minimize isolation and loneliness for older Filipinos in many ways. They assist with using digital methods and videoconferencing technologies to connect with friends and families and continue participating in respective organizations and communities through remote measures. For example, Himan Brown Senior program offers classes and social groups to 700 senior citizens (Finn, 2020). Here, older individuals are taught how to best use digital resources to help maintain their social well-being. Family caregivers also assist their older loved ones access online services for spiritual comfort and support (Armitage and Nellums, 2020). Older Filipinos also now attend holy mass on television, radio, or online because the schedules and frequency have increased several fold.

## Conclusion

Older Filipinos are disproportionately affected by COVID-19. While they constitute less than 8% of the total population, they comprise one-third of all cases and more than half of all deaths related to COVID-19. This underscores what is widely known that older individuals are at a higher risk for COVID-19, with greater morbidity and mortality for this disease. Older Filipinos not only suffer physically but also emotionally, spiritually, and socially. In this unprecedented crisis that developing countries such as the Philippines are not sufficiently equipped to manage, collaborative efforts of the public and private sectors in conjunction with external aids from developed countries and the World Health Organization may help manage the care of our sick older patients adequately. Measures to strengthen the national healthcare infrastructure across the country are imperative in order to more effectively cope with future epidemics.
